# Identifying low acuity Emergency Department visits with a machine learning approach: The low acuity visit algorithms (LAVA)

**DOI:** 10.1111/1475-6773.14305

**Published:** 2024-03-30

**Authors:** Angela T. Chen, Richard S. Kuzma, Ari B. Friedman

**Affiliations:** ^1^ Perelman School of Medicine University of Pennsylvania Philadelphia Pennsylvania USA; ^2^ Health Care Management Department, The Wharton School University of Pennsylvania Philadelphia Pennsylvania USA; ^3^ Leonard Davis Institute of Health Economics University of Pennsylvania Philadelphia Pennsylvania USA; ^4^ Emergency Medicine Department University of Pennsylvania Philadelphia Pennsylvania USA

**Keywords:** algorithms, Emergency Department, ICD‐10, low acuity, machine learning, predictive modeling

## Abstract

**Objective:**

To improve the performance of International Classification of Disease (ICD) code rule‐based algorithms for identifying low acuity Emergency Department (ED) visits by using machine learning methods and additional covariates.

**Data Sources:**

We used secondary data on ED visits from the National Hospital Ambulatory Medical Survey (NHAMCS), from 2016 to 2020.

**Study Design:**

We established baseline performance metrics with seven published algorithms consisting of International Classification of Disease, Tenth Revision codes used to identify low acuity ED visits. We then trained logistic regression, random forest, and gradient boosting (XGBoost) models to predict low acuity ED visits. Each model was trained on five different covariate sets of demographic and clinical data. Model performance was compared using a separate validation dataset. The primary performance metric was the probability that a visit identified by an algorithm as low acuity did not experience significant testing, treatment, or disposition (positive predictive value, PPV). Subgroup analyses assessed model performance across age, sex, and race/ethnicity.

**Data Collection:**

We used 2016–2019 NHAMCS data as the training set and 2020 NHAMCS data for validation.

**Principal Findings:**

The training and validation data consisted of 53,074 and 9542 observations, respectively. Among seven rule‐based algorithms, the highest‐performing had a PPV of 0.35 (95% CI [0.33, 0.36]). All model‐based algorithms outperformed existing algorithms, with the least effective—random forest using only age and sex—improving PPV by 26% (up to 0.44; 95% CI [0.40, 0.48]). Logistic regression and XGBoost trained on all variables improved PPV by 83% (to 0.64; 95% CI [0.62, 0.66]). Multivariable models also demonstrated higher PPV across all three demographic subgroups.

**Conclusions:**

Machine learning models substantially outperform existing algorithms based on ICD codes in predicting low acuity ED visits. Variations in model performance across demographic groups highlight the need for further research to ensure their applicability and fairness across diverse populations.

**What is known on this topic**

- Current methods for identifying low acuity visits to the Emergency Department (ED) are widely used and primarily consist of International Classification of Disease code rule‐based algorithms.
- These existing algorithms have low predictive performance, inconsistent performance across algorithms, and may not capture the complexity of patient conditions.
- Advanced predictive statistical methods and increased data availability suggest the possibility of improving retrospective identification of low acuity ED visits.

**What this study adds**

- Models developed using machine learning methods significantly outperform traditional International Classification of Disease, Tenth Revision code rule‐based algorithms in predicting low acuity Emergency Department visits.
- Inclusion of demographic and clinical variables within these models further enhances prediction accuracy.
- These improved models exhibit more consistent performance across diverse demographic groups.

## 1 INTRODUCTION

A substantial proportion of Emergency Department (ED) visits are thought to be potentially amenable to care in lower‐priced settings.^1^ In the absence of expansions in ED services or improvements in hospital flow,^2^, ^3^ helping patients with these “low acuity” presentations access alternative sites may help alleviate the negative impacts associated with ED crowding, such as increased wait times, strained patient–provider relationships, and excess mortality.^4^, ^5^ Researchers have proposed two potential pathways for reducing low acuity ED visits: improving prevention through increased access to primary care,^6^ and substituting these visits to lower‐cost settings such as urgent care, telemedicine, or primary care.^7^, ^8^ Beyond its relevance to health services research, reducing low acuity ED visits is also of substantial interest to health insurers and public payers, who allocate up to 10% of their expenditures to ED visits.^9^

Studying low acuity ED visits, or interventions which may impact these visits, first requires accurately identifying these cases in available data. The methodology most frequently employed by researchers^10^ stems from Billings et al. (1993), which introduced the ambulatory care‐sensitive conditions algorithm.^11^ This algorithm consists of many International Classification of Disease (ICD) codes, originally developed to identify ED visits and hospitalizations preventable through improved primary care. In practice, however, it has been commonly repurposed to classify low acuity ED visits.^10^ The Billings algorithm and other such ICD code rule‐based approaches have been used or cited in more than 4000 research publications to date.^10^

Despite their widespread use, rule‐based algorithms demonstrate only modest sensitivity and positive predictive value (PPV) in their ability to identify ED visits that could be appropriately managed with resources available in alternative healthcare settings.^10^ Additionally, these algorithms exhibit poor consensus in classifying visits as low acuity, as well as decreased performance when applied to high‐risk subgroups such as older adults, suggesting potential inconsistencies and limitations in their broad application.

Such challenges may stem from the dual use of the original algorithm: identifying both preventable and low acuity visits in data. For instance, the Billings algorithm might correctly identify a severe asthma exacerbation as theoretically preventable through improved primary care, but users of the algorithm might inaccurately label the visit as “low acuity.” We develop a model‐based approach that directly predicts a marker of low acuity in ED visits, to address this second common use for these algorithms.

Increased data availability and advancements in predictive statistical methods hold promise for improving the retrospective identification of low acuity ED visits. Multivariable approaches that use items commonly found in both claims and administrative datasets—such as age, sex, and limited clinical information—may enhance predictive performance while also aligning more closely with ED clinicians' perspectives and best practices.^12^ Machine learning models might further improve accuracy by accommodating interactions between multiple ICD codes—such as in the case of low back pain (typically benign) with urinary retention, which together suggest a spinal cord lesion—while preventing overfitting of the data.^13^ Although methods incorporating more predictors and using data to a fuller capacity have been proposed in the context of insurance risk adjustment,^14^, ^15^ to our knowledge, no study has explored how additional covariates, coupled with a systematic approach to model development using machine learning methods trained on clinical outcomes, might improve upon traditional rule‐based algorithmic performance in identifying low acuity ED visits in data.

In this study, we use data from the National Hospital Ambulatory Medical Survey (NHAMCS) from 2016 to 2019 to train three models—logistic regression, random forest, and extreme gradient boosting (XGBoost)—on a subset of covariates commonly available in claims datasets. Unique for a national dataset, NHAMCS contains ED‐specific variables such as triage codes and ED‐based testing, in addition to variables commonly available in health services research datasets. We validate the performance of each Low Acuity Visit Algorithm (LAVA) on data unavailable during model development (2020 NHAMCS data) and compare the results with the top‐performing ICD code rule‐based low acuity algorithm.^1^, ^10^ We also evaluate model performance in population subgroups who may be more vulnerable to the risks of inappropriate diversion from the ED due to systemic discrimination or higher rates of chronic conditions.

## 2 METHODS

### 2.1 Data

NHAMCS is a nationally representative, annual, repeated cross section of ED visits.^16^ In the stratified sample, approximately 350 EDs nationwide are sampled each year, with approximately 100 charts randomly selected from each, capturing visits from all payers. Data is abstracted from the medical records, creating a comprehensive account of each ED visit. NHAMCS includes variables that are typically available in claims data, such as patient demographics and ICD code diagnoses at discharge, as well as those typically available only in electronic health records, such as the Emergency Severity Index (ESI) triage assignment and the patient's chief complaint at presentation, allowing for the inclusion of clinical signals in constructing a clinically relevant reference standard for this problem. We trained models on the 2016 to 2019 NHAMCS data and validated model performance prospectively on the 2020 data after its release.

We defined the low acuity reference group based on criteria used in prior studies.^10^, ^17^ A visit was classified as *low acuity* if it satisfied all five conditions of the low acuity composite (LAC) outcome: the patient (1) was triaged as nonurgent (ESI level 5) or semi‐urgent (ESI level 4), (2) received fewer than two diagnostic tests, (3) was not admitted to the hospital or transferred to another facility, (4) was not dead on arrival, and (5) did not die in the ED. For the determination of low acuity status, we limited our analyses to observations without any missing components of the LAC outcome.

We selected training variables commonly found in claims and all‐payer databases to increase the relevance of our resulting models for future health services researchers. We compared NHAMCS variables with those available in the Healthcare Cost and Utilization Project's Nationwide Emergency Department Sample (NEDS), the largest all‐payer ED database in the United States.^18^ We aligned variables in NHAMCS with NEDS, identifying the delivery of procedures and other clinical testing using Current Procedural Terminology (CPT) codes. Training variables are listed in Appendix Table 1. Diagnosis codes were coded using ICD‐10 and included at the “category” level, which corresponds to the first three characters of the codes, to avoid overfitting.

### 2.2 Algorithms

We established benchmark performance metrics using the original rule‐based approaches. This involved applying seven algorithms identified in a prior literature review,^10^ each consisting of a list of ICD‐10 codes.^1^, ^11^, ^19^, ^20^, ^21^, ^22^, ^23^ If the primary diagnosis for an observation in NHAMCS matched any of the codes in a particular algorithm, that observation was categorized by the algorithm as “low acuity.” We also accounted for limited interactions between ICD codes that were included in some algorithms, such as Billings.

Next, we trained logistic regression, random forest, and XGBoost models to predict low acuity ED visits as defined by the LAC. These models are standard machine learning approaches known to have good performance for prediction of binary outcomes in tabular data.^24^ Logistic regression, familiar to most clinicians and researchers, provided a strong and interpretable baseline for our multivariable models. We extended this baseline by incorporating two decision tree‐based models—random forests^25^ and gradient‐boosted trees^26^—that permit the mapping of more complex, nonlinear relationships.

Decision trees recursively partition the input data into subsets based on variables that optimize the model's information gain at each split. Random forests employ a technique called “bagging” (bootstrap aggregating) to combine multiple decision trees in parallel, which lessens the risk of overfitting and bolsters the model's generalizability. In contrast, gradient boosting trains decision trees sequentially to correct the mistakes made by previous trees, aiming to minimize prediction errors. Gradient boosting methods are considered state‐of‐the‐art in tabular machine learning, typically outperforming random forests and neural networks for tabular data with a binary outcome.^27^ We used XGBoost,^28^ a popular model that consistently performs well in data science competitions involving tabular data.^24^

### 2.3 Training variables

To explore the impact of including different types of information and to balance parsimony against predictive performance, we trained each of the three models on five sets of covariates. The simplest approach included only age and sex. Age is considered a critical factor as frailty^29^ and multimorbidity^30^ rise with age and place older adults at greater risk of severe disease for a given medical event. Similarly, biological sex may affect resilience to acute health shocks.^31^

The second set of covariates consisted of all ICD‐10 codes present in any of the seven rule‐based algorithms, totaling 306 ICD codes (Appendix Table 2). The third set combined age, sex, and all 306 diagnosis codes. The fourth set included the most comprehensive set of training variables, encompassing covariate set 3 (age, sex, and ICD‐10 codes) and additional clinical features such as procedure and diagnostic test indicators (Appendix Table 1).

For the fifth set of covariates, we identified the 10 most influential variables contributing to model predictions.^32^ In logistic regression, variable importance was determined by the absolute value of the *t*‐statistic for each model parameter. For random forest, importance was assessed by evaluating a variable's impact on model accuracy upon removal. In XGBoost, variables were ranked based on their greatest relative contribution to model performance. Variables that appeared in the top 10 list of at least two of the three models were included in the “influential” covariate subset.

### 2.4 Analysis

We summarized the training data (NHAMCS 2016–2019) and the validation data (NHAMCS 2020) using standard descriptive statistics. This analysis included low acuity status as defined by the LAC, components used to define low acuity visits (such as triage level and death status), and age, sex, and race/ethnicity. We used the independent sample *t*‐test for continuous variables and the Pearson *χ* ^2^ test of independence for categorical variables to assess the statistical significance of differences across datasets. Results were considered significant at *p* < 0.05.

Our primary measure for evaluating model performance was the PPV. PPV represents the probability that a visit identified as low acuity by an algorithm is low acuity according to the LAC definition, and is calculated by dividing the number of true positives (TP) by the sum of true and false positives (FP): $\mathrm{PPV}=\frac{\mathrm{TP}}{\mathrm{TP}+\mathrm{FP}}$. We also recorded sensitivity, specificity, and negative predictive value (NPV) to assess the trade‐offs made between PPV and other performance metrics.^33^, ^34^ To set benchmark estimates for algorithm performance, we applied the seven ICD code rule‐based algorithms to the training data and derived two comparator measures: (1) average PPV across all seven algorithms, and (2) PPV of the best‐performing algorithm. All algorithms, both rule‐based and model‐based, were evaluated against visits categorized as low acuity, as defined by the LAC criteria.

Next, we trained each of the three models (logistic regression, random forest, and XGBoost) on the five sets of covariates, resulting in 15 distinct algorithms. To reduce the risk of overfitting and to obtain more accurate estimates of model performance, we employed tenfold cross‐validation (CV), which involved dividing the dataset into 10 equal parts, training the model on nine parts, and evaluating it on the remaining one. This process was repeated 10 times, each using a different part as the evaluation set. The final estimate of model performance was the average across the 10 “folds.”^14^ If the predicted probability for an observation was more likely to be low acuity than not (*p* > 0.50), we assigned that observation the low acuity label. Finally, we applied all 15 trained models to the validation dataset (NHAMCS 2020) for an out‐of‐sample evaluation of model performance.

We illustrated the trade‐off between PPV and sensitivity using precision‐recall curves. These curves plot PPV (precision) on the *y*‐axis and sensitivity (recall) on the *x*‐axis. While our primary analysis utilized a 0.5 probability threshold for binary classification, the precision‐recall curves showcase how PPV and sensitivity change with varying probability thresholds. We focused on the two best‐performing machine learning models, each trained on three sets of variables, for a total of six precision‐recall curves. The variable sets included: (1) all ICD‐10 codes, enabling a comparison with rule‐based approaches; (2) all variables, which we hypothesized to yield models with the highest PPV; and (3) the “influential subset” of variables, offering insights into how models perform with a reduced set of features.

To assess for differences in model performance across demographic groups, we also applied each trained model to demographic subsets of the NHAMCS 2020 data based on age, sex, and race/ethnicity. For the age analysis, patients were categorized into three groups: 18–44 years, 45–64 years, and over 65 years. Sex was classified as male and female. Race and ethnicity consisted of non‐Hispanic White, non‐Hispanic Black, and Hispanic. We excluded Non‐Hispanic “Other” patients from this analysis, as this category captures a wide range of ethnic and racial identities and accounts for less than 5% of the overall sample size.

Due to the limited presence of biologically plausible mechanisms linking race and ethnicity to the relationship between ICD codes and the low acuity composite outcomes, and because of the potential for perpetuating race‐based health inequities,^35^ we excluded race/ethnicity as a covariate in our primary models. The exclusion was driven by concerns that Black or Hispanic individuals may be systematically assigned less acute triage scores,^36^ thereby increasing the likelihood of categorizing their visits as low acuity. In such cases, inclusion of the race/ethnicity variable could inadvertently raise the threshold for allocating clinical resources to these patients. However, recognizing that inclusion of race and ethnicity might improve model predictive performance,^37^ we conducted a sensitivity analysis in which both primary and subgroup analyses were repeated using models trained with the inclusion of the race/ethnicity variable.

We used R version 4.1.3 for analyses.^38^ We did not weight analyses because the goal was to train models for inference on individual cases. We adhered to the Transparent Reporting of a Multivariable Prediction Model for Individual Prognosis or Diagnosis (TRIPOD) guidelines (Appendix Table 3).^39^ Since analyses were conducted using publicly available and de‐identified observational data, this study did not meet the criteria for human subjects research and was exempt from IRB review.

## 3 RESULTS

The NHAMCS 2016–2019 training data included 53,074 observations, and the NHAMCS 2020 validation data included 9542 observations (Table 1). Of these, 27.0% of the visits in 2016–2019 and 22.4% in 2020 were classified as low acuity based on the LAC criteria (*p* < 0.001). The median age of patients was younger in the 2016–2019 data compared to the 2020 data (35 years [IQR 19–56] vs. 40 years [IQR 22–60], *p* < 0.001), and NHAMCS 2016–2019 had a lower proportion of male patients (45.6% vs. 47.8% in 2020, *p* < 0.001). Patients in the training dataset had fewer diagnostic services performed (2016–2019: median 2 [IQR 1–3], vs. 2020: median 3 [IQR 1–3], *p* < 0.001), a lower proportion of patients triaged as “urgent” and “emergent” (48.6% and 12.9%, vs. 51.6% and 15.6% in 2020; *p <* 0.001), and a lower rate of hospital admissions from the ED compared to the validation dataset (10.1% vs. 13.6% in 2020; *p* < 0.001).

**TABLE 1:** Summary of National Hospital Ambulatory Medical Survey data, years 2016–2019 versus 2020.

	NHAMCS 2016–2019 (*N* = 53,074)	NHAMCS 2020 (*N* = 9542)	*p* value
Low acuity^a^	14,336 (27.0%)	2141 (22.4%)	<0.001
Age, median [IQR], years	35 [19–56]	40 [22–60]	<0.001
Male	24,182 (45.6%)	4564 (47.8%)	<0.001
Race/ethnicity			
Non‐Hispanic White	29,458 (55.5%)	5357 (56.1%)	0.10
Non‐Hispanic Black	13,060 (24.6%)	2237 (23.4%)	
Hispanic	8444 (15.9%)	1561 (16.4%)	
Non‐Hispanic Other	2112 (4.0%)	387 (4.1%)	
Total number of diagnoses,^b^ median [IQR]	2 [1–3]	2 [1–3]	<0.001
Total number of diagnostic services, median [IQR]	2 [1–6]	3 [1–6]	<0.001
Triage level			
Immediate	621 (1.2%)	99 (1.0%)	<0.001
Emergent	6873 (12.9%)	1491 (15.6%)	
Urgent	25,796 (48.6%)	4924 (51.6%)	
Semi‐urgent	17,047 (32.1%)	2629 (27.6%)	
Nonurgent	2737 (5.2%)	399 (4.2%)	
Admitted to hospital	5351 (10.1%)	1301 (13.6%)	<0.001
Dead on arrival	3 (0.0%)	4 (0.0%)	0.01
Died in the ED	57 (0.1%)	17 (0.2%)	0.09

Abbreviations: ED, Emergency Department; IQR, interquartile range; NHAMCS, National Hospital Ambulatory Medical Care Survey.

^a^ Defined by the following criteria: the ED visit (1) was triaged as nonurgent (Emergency Severity Index [ESI] 5) or semi‐urgent (ESI 4), (2) received fewer than two diagnostic tests, (3) was not admitted to the hospital or transferred to another facility, (4) was not dead on arrival, and (5) did not die in the ED.

^b^ Up to five diagnoses reported in NHAMCS data.

**FIGURE 1:**
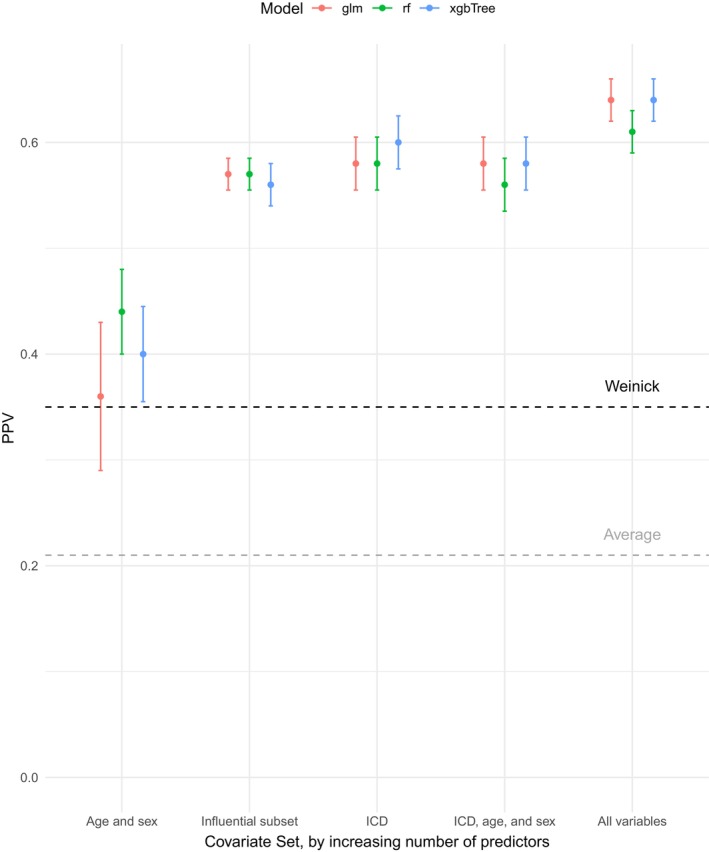
Model performance on National Hospital Ambulatory Medical Survey 2020 data, measured by positive predictive value (PPV). This figure illustrates the performance, quantified by PPV, of all 15 models on the validation dataset. Logistic regression (*glm*), random forest (*rf*), and XGBoost (*xgbTree*) were each trained on five distinct covariate sets: (1) age and sex, (2) an “influential subset” of variables (age, any imaging, CBC, CT scan, flu test, IV fluids, pregnancy test, urinalysis, and urine culture), (3) the International Classification of Disease, Tenth Revision (ICD‐10) codes included in any of the original seven algorithms (totaling 306 codes), (4) ICD‐10 codes, age, and sex, and (5) all variables. The black dashed line marks the performance of the Weinick algorithm, which was the best among the original ICD code rule‐based algorithms, and the gray dashed line marks the average PPV of these seven rule‐based algorithms.

Figure 1 illustrates the model performance, as measured by PPV, of logistic regression, random forest, and XGBoost models trained on different sets of predictors. The “influential subset” comprised of nine variables: age, any imaging, CBC, CT scan, flu test, IV fluids, pregnancy test, urinalysis, and urine culture (Appendix Table 4). Figure 1 also compares the performance of the machine learning models to the best‐performing ICD code rule‐based approach. Models trained on only age and sex had the lowest PPV of all candidate models, but performed at least as well as the best‐performing rule‐based algorithm.^1^ For example, the random forest model trained on age and sex demonstrated a 26% increase in PPV (random forest 0.44; 95% CI [0.40, 0.48]); Weinick algorithm 0.35 (95% CI [0.33, 0.36]; Table 2). Models trained exclusively on ICD codes also outperformed the original algorithms; XGBoost achieved a PPV of 0.60 (95% CI [0.57, 0.62]), while the best and average rule‐based algorithm had PPVs of 0.35 (95% CI [0.33, 0.36]) and 0.21 (95% CI [0.14, 0.28]), respectively, representing relative increases of 71% and 186%. Logistic regression and XGBoost achieved PPV values of 0.64 (95% CI [0.62, 0.66]) when trained all covariates, representing an 83% increase over the best rule‐based algorithm.

**TABLE 2:** Model performance on the validation sample, by model and covariate set.

Model	Covariate set	Sensitivity, 95% CI	Specificity, 95% CI	PPV, 95% CI	NPV, 95% CI
Weinick^a^	ICD	0.57 [0.55, 0.59]	0.69 [0.68, 0.70]	0.35 [0.33, 0.36]	0.85 [0.84, 0.86]
Average^b^	ICD	0.24 [0.10, 0.38]	0.78 [0.71, 0.86]	0.21 [0.14, 0.28]	0.78 [0.76, 0.81]
Logistic regression	Age and sex	0.03 [0.02, 0.04]	0.98 [0.98, 0.99]	0.36 [0.29, 0.43]	0.78 [0.77, 0.79]
	ICD^c^	0.37 [0.35, 0.39]	0.92 [0.92, 0.93]	0.58 [0.56, 0.61]	0.83 [0.83, 0.84]
	ICD, age, and sex	0.39 [0.37, 0.41]	0.92 [0.91, 0.93]	0.58 [0.56, 0.61]	0.84 [0.83, 0.85]
	Influential subset^d^	0.81 [0.79, 0.82]	0.82 [0.81, 0.83]	0.57 [0.55, 0.58]	0.94 [0.93, 0.94]
	All variables	0.79 [0.77, 0.81]	0.87 [0.86, 0.88]	0.64 [0.62, 0.66]	0.94 [0.93, 0.94]
Random forest	Age and sex	0.12 [0.11, 0.14]	0.96 [0.95, 0.96]	0.44 [0.40, 0.48]	0.79 [0.78, 0.80]
	ICD	0.37 [0.35, 0.39]	0.92 [0.92, 0.93]	0.58 [0.56, 0.61]	0.84 [0.83, 0.84]
	ICD, age, and sex	0.42 [0.40, 0.44]	0.90 [0.90, 0.91]	0.56 [0.53, 0.58]	0.84 [0.84, 0.85]
	Influential subset	0.77 [0.75, 0.78]	0.84 [0.83, 0.84]	0.57 [0.56, 0.59]	0.93 [0.92, 0.93]
	All variables	0.75 [0.73, 0.77]	0.86 [0.85, 0.87]	0.61 [0.59, 0.63]	0.92 [0.92, 0.93]
XGBoost	Age and sex	0.10 [0.09, 0.12]	0.96 [0.95, 0.96]	0.40 [0.36, 0.45]	0.79 [0.78, 0.80]
	ICD	0.34 [0.32, 0.36]	0.93 [0.93, 0.94]	0.60 [0.57, 0.62]	0.83 [0.82, 0.84]
	ICD, age, and sex	0.38 [0.36, 0.40]	0.92 [0.91, 0.93]	0.58 [0.56, 0.61]	0.84 [0.83, 0.85]
	Influential subset	0.85 [0.83, 0.86]	0.81 [0.80, 0.81]	0.56 [0.54, 0.58]	0.95 [0.94, 0.95]
	All variables	0.78 [0.76, 0.80]	0.87 [0.86, 0.88]	0.64 [0.62, 0.66]	0.93 [0.93, 0.94]

Abbreviations: ICD, International Classification of Disease; NPV, negative predictive value; PPV, positive predictive value; XGBoost, extreme gradient boosting.

^a^ The top‐performing algorithm among seven published rule‐based algorithms based on ICD‐10 codes.

^b^ The average across seven published rule‐based algorithms based on ICD‐10 codes.

^c^ The set of ICD‐10 codes included in any of the original seven algorithms; 306 codes total.

^d^ Includes age, any imaging, CBC, CT scan, flu test, IV fluids, pregnancy test, urinalysis, and urine culture. See Section 2 and Appendix Table 4 for details on variable selection.

Model performance improved with the inclusion of more covariates, and relative performance of models varied depending on the covariates included. While random forest had the highest PPV when trained on only two predictors, it had a lower PPV than both logistic regression and XGBoost when all covariates were included. Detailed model performance statistics, including sensitivity, specificity, and NPV, are reported in Table 2. Calibration plots, which provide a more complete view of model performance by plotting the predicted model probabilities against the true class probabilities of the data, are included in Appendix Figure 1. Logistic regression and XGBoost models demonstrated superior calibration compared to random forest models.

**FIGURE 2:**
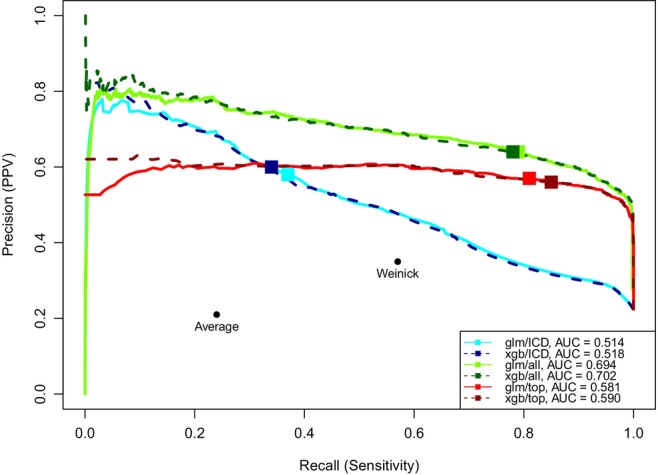
Precision‐recall (PPV‐sensitivity) curves for selected logistic and extreme gradient boosting (XGBoost) models. This figure presents the trade‐off between positive predictive value (PPV) and sensitivity for six models at varying probability thresholds. Sensitivity is plotted on the *x*‐axis and PPV on the *y*‐axis. Displayed models include logistic regression (*glm*; solid lines) and XGBoost (*xgb*; dashed lines), each trained on (1) the International Classification of Disease, Tenth Revision (ICD‐10) codes from any original algorithm (totaling 306 codes; *icd*, blue lines), (2) all variables (*all*, green lines), and (3) the “influential subset” of variables (*top*, red lines). The downward trend of lines indicates the trade‐off between measures, as sensitivity increases (by lowering the threshold for low acuity labeling), PPV decreases. Square points on each model's precision‐recall curve represent sensitivity and PPV measures at the 0.5 probability threshold, as reported in Table 2. A model with perfect prediction would have an area under the curve (AUC) of 1.0; the *xgb/all* model exhibits the highest performance with an AUC of 0.702. The ICD code rule‐based algorithms, due to their deterministic nature, are depicted as individual points below and to the left of all curves, indicating their lower PPV and sensitivity compared to the trained models.

Precision‐recall curves (Figure 2) compare the performance of the logistic regression and XGBoost models, which were the two models with the highest PPV when trained on the full set of variables. These curves illustrate the PPV‐sensitivity trade‐off; by adjusting the probability threshold at which instances are classified as positive or negative by the machine learning models, PPV may improve at the expense of reduced sensitivity, or vice versa. The PPV and sensitivity values for these models at the 0.5 probability threshold, as reported in Table 2, are represented as square points. Overall, performance of logistic regression (solid lines) and XGBoost (dashed lines) was comparable. The area under the curve (AUC) values ranged from 0.514 (logistic regression trained on ICD codes only) to 0.702 (XGBoost trained on all predictors). Due to their deterministic nature, rule‐based algorithms are represented as single points below and to the left of all curves, outperformed by all trained models.

We standardized four models from Figure 2 for reference in future research. Two models, the Low Acuity Validation Algorithm—Logistic regression (LAVA‐L) and the Low Acuity Validation Algorithm—XGBoost (LAVA‐X), were trained on all variables. Though we used a default 0.5 probability in our analyses, alternative thresholds can be employed (details in Appendix Table 5). LAVA‐L‐sub and LAVA‐X‐sub are counterparts of the former models and were trained on the “influential subset” of nine variables.

**FIGURE 3:**
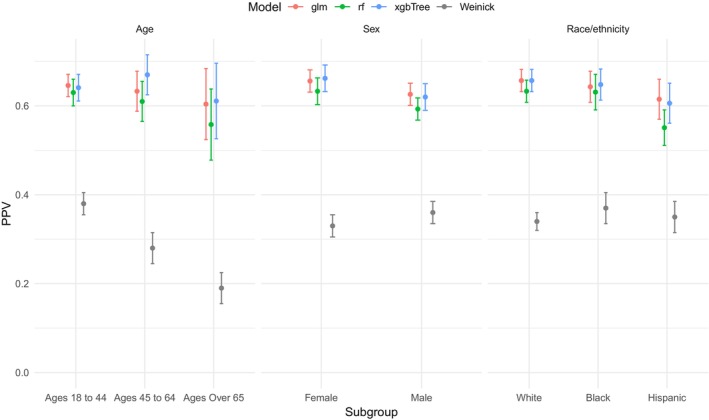
Subgroup performance by age, sex, and race/ethnicity of models trained on all variables. This figure displays the positive predictive value (PPV, *y*‐axis) performance of logistic regression (*glm*), random forest (*rf*), and extreme gradient boosting (XGBoost) (*xgbTree*) models trained on all variables and applied to different demographic subgroups, as specified on the *x*‐axis. The performance of the Weinick algorithm, the most effective International Classification of Disease code rule‐based algorithm, is plotted in gray for comparison.

Model performance varied by demographic subgroup (Figure 3). The logistic regression, random forest, and XGBoost models, trained on all predictors, varied by less than 0.10 in PPV across subgroups defined by age, sex, and race/ethnicity. Additionally, model performance surpassed the best rule‐based approach in all subgroups.

Detailed results of sensitivity analyses using models trained on the race/ethnicity variable are reported in Appendix Table 6. After training all three models on the complete covariate set, race/ethnicity emerged among the top 10 most important variables only for the random forest model (Appendix Table 7). Visual comparisons of PPV outcomes between the primary and sensitivity analyses are presented in Appendix Figures 2 and 3. Inclusion of the race/ethnicity variable did not significantly alter point estimates or their interpretations.

## 4 DISCUSSION

In this study, we revisited rule‐based approaches to identifying low acuity ED visits by broadening the information available to the model, using flexible models that allow for interactions between variables without overfitting, systematically fitting models to training data with a consistent definition of low acuity, and validating on holdout data. We evaluated the performance of logistic regression, random forest, and XGBoost algorithms using NHAMCS data, comparing them to ICD code rule‐based approaches. Our findings support our hypothesis that multivariable machine learning improves upon widely used rule‐based approaches in identifying low acuity ED visits.

The model‐based algorithms outperformed the rule‐based algorithms in all analyses. While 35% (95% CI [33%, 36%]) of ED visits identified as low acuity by the best‐performing rule‐based algorithm^1^ met the criteria of the LAC outcome, 64% (95% CI [62%, 66%]) of ED visits identified as low acuity by XGBoost trained on all covariates met the composite criteria, which was an 83% increase in PPV. Relative improvement over all other rule‐based algorithms was even more pronounced.

In our subgroup analyses we found consistent model performance among the age groups of 18 to 44 and 45 to 64 and a modest drop‐off in performance for individuals aged 65 and over. This aligns with our hypothesis regarding the geriatric population, as older adults present with a broader range of diagnoses and symptoms that pose challenges for accurate prediction of low acuity ED visits. Multivariable approaches which allow for the impact of each ICD code to vary by age mitigated the decline in performance for this high‐risk group compared to the rule‐based algorithmic approaches. These results suggest that including age—which proxies for biological age, frailty, multimorbidity, and cognitive impairment—can help maintain model performance among older adults.

Our study also revealed variations in model performance based on race and ethnicity. The models demonstrated better predictive ability for patients from White and Black populations compared to those with Hispanic identities, who were less represented in the data (Table 1). The consistent results of our sensitivity analysis with models trained on race and ethnicity as a predictor suggest that the additional predictive power of race and ethnicity may be marginal when ICD‐10 diagnosis codes and clinical testing information are also available.

The machine learning, model‐based approach presented in this study offers a more performant alternative to traditional rule‐based methods. Despite statistically significant differences in the distributions of most variables between the NHAMCS 2016–2019 and 2020 datasets (Table 1), the performance of the logistic regression model trained on all variables remained relatively stable, with only a 5% decrease in PPV (2016–2019 PPV = 0.66; 95% CI [0.66, 0.67]; Appendix Table 8 vs. 2020 PPV = 0.64; 95% CI [0.62, 0.66]). Given the historic impact of the COVID‐19 pandemic on the magnitude and composition of ED visits,^40^ the strong 2020 model performance raises confidence in the out‐of‐sample performance of these algorithms. In comparison, the Weinick algorithm's PPV declined by 0.06 between time periods (2016–2019 PPV = 0.41; 95% CI [0.40, 0.41] vs. 2020 PPV = 0.35; 95% CI [0.33, 0.36]).

While PPV was our primary outcome, researchers may prioritize sensitivity in certain scenarios. The results reported in Table 2 reflect a cutoff threshold of 0.5, where observations with a predicted probability above 0.5 were labeled “low acuity.” However, the precision‐recall curves (Figure 2) demonstrate that researchers can achieve a range of PPV and sensitivity values for each model by choosing different thresholds, providing flexibility over established, ICD code rule‐based methods.

We provide four standard models for use in future health services research. Each model's probability threshold for determining a positive instance can be modified to match researchers' goals (Appendix Table 5). For example, the LAVA‐L model with a threshold of 0.8 is suitable for applications where each low acuity visit identified should be reliably low acuity (78% PPV), while accepting the trade‐off of identifying only 12% of all low acuity ED visits. By contrast, the LAVA‐X algorithm with a threshold of 0.3 captures most low acuity visits (94% sensitivity), while modestly trading off PPV: 57% of visits identified as low acuity meet the low acuity composite criteria. LAVA‐L‐sub and LAVA‐X‐sub can be used when only a limited number of variables are available for analysis. All four models are available in R and accessible on GitHub at https://www.github.com/angela-t-chen/lava.ed.

Importantly, although the machine learning methods presented in this study offer significant improvements over current approaches, they are intended for retrospective applications aimed at enhancing research quality. A perfect classifier would have an AUC of 1 in the precision‐recall curve; our models reached the highest AUC of 0.702, indicating room for improvement. Limitations of model performance preclude the application of these models in clinical triage or retrospective payment denial. Development of models with actual implications for patient well‐being and healthcare practices necessitates additional testing, validation, and model refinement. Furthermore, such efforts should be conducted with a focus on defined objectives, such as ensuring transparency in model development, enhancing patient safety, and promoting equity.^13^

To the best of our knowledge, this study represents the first attempt to propose a systematic, multivariable approach as an alternative for identifying low acuity ED visits in claims or administrative datasets. While our findings align with previous research that identifies younger age as an important determinant of low acuity status,^41^, ^42^, ^43^ our primary objective was not to identify specific predictors that are particularly influential in classifying such visits. Instead, we propose a more nuanced approach. Efforts are already underway to employ machine learning in reevaluating traditional prediction methods, particularly in the context of risk scores.^13^, ^14^, ^15^, ^44^ We hope that our work serves as another example of using more complex—yet accessible—statistical tools to improve existing health services research methods.

We note several limitations of this study. We have not yet tested these algorithms beyond NHAMCS data, which may limit the generalizability of our findings. However, we ensured that our models were trained on predictor variables typically available in claims and administrative datasets, and we evaluated the models on a separate dataset that was released after the models were developed. This dataset differed slightly from the training data, likely due to the impact of COVID‐19, and is unlikely to lead to spectrum bias given the modest differences. Further work remains to validate these algorithms in other contexts, such as multisystem electronic health record datasets.

We chose a conservative definition of low acuity from existing literature that matches the approximate capabilities of many ED alternatives. Studies of clinical sites with capabilities to conduct more advanced testing, such as urgent care clinics with CT scanners, may need models developed using a novel definition of low or intermediate acuity. Similarly, our results may not match well to retail clinics, whose clinical capabilities are far more limited than primary care, or telemedicine, where even a single laboratory test may require the patient to have a separate visit to an outpatient lab.

Additionally, while machine learning models like XGBoost may offer superior predictive performance, their complexity can hinder interpretability and practical implementation compared to simpler models such as logistic regression. Our results indicate that logistic regression and XGBoost exhibit comparable performance (Figure 2). Therefore, in the retrospective identification of low acuity visits in ED data, using logistic regression may be justified. To further facilitate reproducibility, we have included the coefficients of the logistic models in Appendix Table 9.

We demonstrate that a model‐based approach using readily available ED data can outperform ICD code rule‐based algorithms in identifying low acuity ED visits. These models achieved higher PPVs and exhibited better performance both overall and across demographic subgroups, compared to commonly used algorithms. Thus, multivariable, model‐based approaches offer a flexible and effective alternative to relying solely on ICD codes. While we have validated these findings out of sample in NHAMCS, these models require validation and careful testing of their applications in other settings before use. Ultimately, our findings have important implications for improving the accuracy of low acuity ED visit identification, which can help inform resource allocation and optimize patient care.

## Supporting information


**Appendix Table 1:** All training variables.
**Appendix Table 2:** International Classification of Disease codes included in model training.
**Appendix Table 3:** TRIPOD checklist.
**Appendix Table 4:** Influential features affecting model predictions.
**Appendix Figure 1:** Calibration plots, primary analysis.
**Appendix Table 5:** Model performance with varying classification thresholds.
**Appendix Table 6:** Sensitivity analysis, model performance on the validation sample.
**Appendix Table 7:** Sensitivity analysis, 10 most influential features affecting model predictions.
**Appendix Figure 2:** Comparison of primary and sensitivity analysis model performance, positive predictive value.
**Appendix Figure 3:** Sensitivity analysis, subgroup performance of model trained on all variables (including race/ethnicity).
**Appendix Table 8:** Model performance on training sample, by model and variable set.
**Appendix Table 9:** Logistic regression model coefficients.
