# Supplementary material for: Identifying low acuity Emergency Department visits with a machine learning approach: The low acuity visit algorithms (LAVA)
Source: Health Serv Res. 2024 Mar 30;59(4):e14305. doi: 10.1111/1475-6773.14305 (PMC11249839; doi:10.1111/1475-6773.14305)
Supplement: Supplementary file 1 — Appendix Table 1: All training variables. Appendix Table 2: International Classification of Disease codes included in model training. Appendix Table 3: TRIPOD checklist. Appendix Table 4: Influential features affecting model predictions. Appendix Figure 1: Calibration plots, primary analysis. Appendix Table 5: Model performance with varying classification thresholds. Appendix Table 6: Sensitivity analysis, model performance on the validation sample. Appendix Table 7: Sensitivity analysis, 10 most influential features affecting model predictions. Appendix Figure 2: Comparison of primary and sensitivity analysis model performance, positive predictive value. Appendix Figure 3: Sensitivity analysis, subgroup performance of model trained on all variables (including race/ethnicity). Appendix Table 8: Model performance on training sample, by model and variable set. Appendix Table 9: Logistic regression model coefficients. [file HESR-59-0-s001.docx]

Identifying Low Acuity Emergency Department Visits with a Machine Learning Approach: The Low Acuity Visit Algorithms (LAVA)

Appendix

Table of Contents

[Appendix Table 1: All training variables^a,b^ 2](#_Toc157427200)

[Appendix Table 2: ICD codes included in model training 3](#_Toc157427201)

[Appendix Table 3: TRIPOD Checklist 4](#_Toc157427202)

[Appendix Table 4: Influential features affecting model predictions^a^ 6](#_Toc157427203)

[Appendix Figure 1: Calibration plots, primary analysis 7](#_Toc157427204)

[Appendix Table 5. Model Performance with varying classification thresholds 10](#_Toc157427205)

[Appendix Table 6. Sensitivity analysis, model performance on the validation sample 11](#_Toc157427206)

[Appendix Table 7. Sensitivity analysis, ten most influential features affecting model predictions^a^ 12](#_Toc157427207)

[Appendix Figure 2. Comparison of primary and sensitivity analysis model performance, PPV 13](#_Toc157427208)

[Appendix Figure 3. Sensitivity analysis, subgroup performance of model trained on all variables (including race/ethnicity) 14](#_Toc157427209)

[Appendix Table 8. Model performance on training sample, by model and variable set 15](#_Toc157427210)

[Appendix Table 9. Logistic regression model coefficients 16](#_Toc157427211)

# **Appendix Table 1: All training variables^a,b^**

| NHAMCS variable | Description | NHAMCS variable | Description |
| --- | --- | --- | --- |
| *age* | Patient age in years | *endoint* | Endotracheal intubation |
| *sex* | Sex (Female/Male) | *flutest* | Influenza test |
| *diag1* | Diagnosis #1 in ICD-10 | *glucose* | Glucose, serum |
| *anyimage* | Any imaging | *hivtest* | HIV test |
| *bac* | Blood alcohol concentration | *incdrain* | Incision & drainage (I&D) |
| *bladcath* | Bladder catheter | *ivfluids* | IV fluids |
| *bloodcx* | Blood culture | *lactate* | Lactate |
| *bnp* | Brain natriuretic peptide | *lft* | Liver enzymes/Hepatic function panel |
| *bpap* | BPAP/CPAP | *lumbar* | Lumbar puncture (LP) |
| *buncreat* | Creatinine/renal function panel | *mri* | MRI |
| *cardenz* | Cardiac enzymes | *nebuther* | Nebulizer therapy |
| *catscan* | CT scan (any) | *othimage* | Other imaging |
| *cbc* | Complete blood count | *pregtest* | Pregnancy/HCG test |
| *centline* | Central line | *pttinr* | Prothrombin time (PT/PTT/INR) |
| *cpr* | CPR | *skinadh* | Skin adhesives |
| *ctab* | CT scan – abdomen/pelvis | *suture* | Suturing/staples |
| *ctchest* | CT scan – chest | *toxscren* | Toxicology screen |
| *cthead* | CT scan – head | *ultrasnd* | Ultrasound |
| *ctother* | CT scan - other | *urine* | Urinalysis (UA) or urine dipstick |
| *ddimer* | D-dimer | *urinecx* | Culture, urine |
| *edhiv* | HIV infection/AIDS | *woundcx* | Culture, wound |
| *electrol* | Electrolytes | *xray* | X-ray |

^a^ All variables other than *age*, *sex*, and *diag1* report whether the diagnostic service or procedure was performed (Y/N). These variables do *not* report test or procedure results.

^b^ Clinical variables (*anyimage* to *xray*) were selected via a manual review aligning chart review-based variables in NHAMCS with those found in the Healthcare Cost and Utilization Project’s Nationwide Emergency Department Sample (NEDS). Procedures and other clinical testing were identified using Current Procedural Terminology (CPT) codes. CPT codes that were available but are not typically billed separately (e.g., electrocardiograms) were excluded.

# **Appendix Table 2: ICD codes included in model training**

| A02 A04 A05 A07 A08 A09 A37 A38 A54 A56 A63 A64 A69 A74  B07 B15 B16 B17 B20 B30 B34 B85 B86 B97 B99  C44  D48 D50 D53 D57  E03 E04 E05 E10 E11 E13 E16 E51 E55 E61 E66 E83 E86 E87 E89  F01 F03 F10 F11 F32 F33 F34 F39 F40 F41 F43 F45 F50 F60  G35 G40 G43 G44 G47 G56 G62  H00 H01 H02 H10 H11 H16 H40 H60 H61 H65 H66 H67 H69 H91 H93  I09 I10 I11 I12 I13 I15 I16 I20 I24 I25 I49 I50 I67 I70 I73 I80 I82 I83 I86 I87 I95  J00 J01 J02 J03 J04 J05 J06 J09 J10 J11 J12 J15 J16 J18 J20 J21 J30 J31 J32 J34 J35 J40 J41 J42 J43 J44 J45 J47 J81 J84  K00 K01 K02 K03 K04 K05 K06 K08 K09 K11 K12 K13 K14 K21 K25 K26 K27 K29 K30 K31 K35 K52 K57 K58 K59 K70  L01 L02 L03 L04 L05 L08 L20 L22 L23 L60 L72 L89 L97 L98  M10 M16 M17 M19 M20 M21 M25 M27 M47 M53 M54 M60 M62 M65 M67 M70 M71 M75 M76 M77 M79 M99  N10 N12 N13 N15 N28 N30 N34 N36 N39 N41 N45 N48 N70 N71 N72 N73 N75 N76 N84 N87  O23 O24 Q34 Q64 Q89  R00 R04 R05 R06 R07 R10 R11 R21 R30 R31 R32 R47 R50 R51 R52 R53 R56 R62 R63  S00 S01 S02 S03 S05 S09 S10 S13 S20 S21 S22 S23 S29 S30 S31 S32 S33 S39 S40 S41 S42 S43 S46 S50 S51 S52 S53 S56 S60 S61 S62 S63 S66 S70 S71 S73 S76 S80 S81 S82 S83 S86 S90 S91 S92 S93 S96 S99  T14 T67 T75 T78 T83  Z01 Z09 Z23 Z29 Z41 Z43 Z47 Z48 Z59 Z63 Z72 Z73 Z74 Z75 Z76 |
| --- |

# **Appendix Table 3: TRIPOD Checklist**

| **Section/Topic** | **Item** |  | **Checklist Item** | **Page** |
| --- | --- | --- | --- | --- |
| **Title and abstract** | | | | |
| Title | 1 | D;V | Identify the study as developing and/or validating a multivariable prediction model, the target population, and the outcome to be predicted. | 1 |
| Abstract | 2 | D;V | Provide a summary of objectives, study design, setting, participants, sample size, predictors, outcome, statistical analysis, results, and conclusions. | 2 |
| **Introduction** | | | | |
| Background and objectives | 3a | D;V | Explain the medical context (including whether diagnostic or prognostic) and rationale for developing or validating the multivariable prediction model, including references to existing models. | 5 |
|  | 3b | D;V | Specify the objectives, including whether the study describes the development or validation of the model or both. | 6 |
| **Methods** | | | | |
| Source of data | 4a | D;V | Describe the study design or source of data (e.g., randomized trial, cohort, or registry data), separately for the development and validation data sets, if applicable. | 7 |
|  | 4b | D;V | Specify the key study dates, including start of accrual; end of accrual; and, if applicable, end of follow-up. | 7 |
| Participants | 5a | D;V | Specify key elements of the study setting (e.g., primary care, secondary care, general population) including number and location of centres. | 7 |
|  | 5b | D;V | Describe eligibility criteria for participants. | 8 |
|  | 5c | D;V | Give details of treatments received, if relevant. | N/A |
| Outcome | 6a | D;V | Clearly define the outcome that is predicted by the prediction model, including how and when assessed. | 8 |
|  | 6b | D;V | Report any actions to blind assessment of the outcome to be predicted. | N/A |
| Predictors | 7a | D;V | Clearly define all predictors used in developing or validating the multivariable prediction model, including how and when they were measured. | 10 |
|  | 7b | D;V | Report any actions to blind assessment of predictors for the outcome and other predictors. | N/A |
| Sample size | 8 | D;V | Explain how the study size was arrived at. | 7 |
| Missing data | 9 | D;V | Describe how missing data were handled (e.g., complete-case analysis, single imputation, multiple imputation) with details of any imputation method. | 8 |
| Statistical analysis methods | 10a | D | Describe how predictors were handled in the analyses. | 10 |
|  | 10b | D | Specify type of model, all model-building procedures (including any predictor selection), and method for internal validation. | 9 |
|  | 10c | V | For validation, describe how the predictions were calculated. | 12 |
|  | 10d | D;V | Specify all measures used to assess model performance and, if relevant, to compare multiple models. | 11 |
|  | 10e | V | Describe any model updating (e.g., recalibration) arising from the validation, if done. | 12 |
| Risk groups | 11 | D;V | Provide details on how risk groups were created, if done. | 10 |
| Development vs. validation | 12 | V | For validation, identify any differences from the development data in setting, eligibility criteria, outcome, and predictors. | 7 |
| **Results** | | | | |
| Participants | 13a | D;V | Describe the flow of participants through the study, including the number of participants with and without the outcome and, if applicable, a summary of the follow-up time. A diagram may be helpful. | 13 |
|  | 13b | D;V | Describe the characteristics of the participants (basic demographics, clinical features, available predictors), including the number of participants with missing data for predictors and outcome. | 13 |
|  | 13c | V | For validation, show a comparison with the development data of the distribution of important variables (demographics, predictors and outcome). | 13 |
| Model development | 14a | D | Specify the number of participants and outcome events in each analysis. | 13 |
|  | 14b | D | If done, report the unadjusted association between each candidate predictor and outcome. | N/A |
| Model specification | 15a | D | Present the full prediction model to allow predictions for individuals (i.e., all regression coefficients, and model intercept or baseline survival at a given time point). | A8 |
|  | 15b | D | Explain how to the use the prediction model. | A8 |
| Model performance | 16 | D;V | Report performance measures (with CIs) for the prediction model. | 14 |
| Model-updating | 17 | V | If done, report the results from any model updating (i.e., model specification, model performance). | N/A |
| **Discussion** | | | | |
| Limitations | 18 | D;V | Discuss any limitations of the study (such as nonrepresentative sample, few events per predictor, missing data). | 20 |
| Interpretation | 19a | V | For validation, discuss the results with reference to performance in the development data, and any other validation data. | 18 |
|  | 19b | D;V | Give an overall interpretation of the results, considering objectives, limitations, results from similar studies, and other relevant evidence. | 19 |
| Implications | 20 | D;V | Discuss the potential clinical use of the model and implications for future research. | 19 |
| **Other information** | | | | |
| Supplementary information | 21 | D;V | Provide information about the availability of supplementary resources, such as study protocol, Web calculator, and data sets. | 7 |
| Funding | 22 | D;V | Give the source of funding and the role of the funders for the present study. | 1 |

# **Appendix Table 4: Influential features affecting model predictions^a^**

| Logistic regression | Random Forest | XGBoost |
| --- | --- | --- |
| 1. **CBC^b^** | 1. **CBC** | 1. **CBC** |
| 1. **Urinalysis** | 1. **Age** | 1. **Urinalysis** |
| 1. **Flu test** | 1. **Urinalysis** | 1. **IV fluids** |
| 1. **IV fluids** | 1. **IV fluids** | 1. **CT scan** |
| 1. **Pregnancy test** | 1. **CT scan** | 1. **Any imaging** |
| 1. Ultrasound | 1. Sex | 1. **Pregnancy test** |
| 1. ICD M25 (Other joint disorders) | 1. **Any imaging** | 1. **Age** |
| 1. **Age** | 1. **Flu test** | 1. **Flu test** |
| 1. ICD S93 (Dislocation and sprain of joints and ligaments at ankle, foot and toe level) | 1. **Pregnancy test** | 1. **Urine culture** |
| 1. X-ray | 1. **Urine culture** | 1. Glucose, serum |

^a^ How variable importance is determined:

- Logistic regression: the absolute value of the t-statistic for each model parameter
- Random forest: removal results in greatest decreases in model accuracy
- XG Boost: greatest relative contribution of each variable

^b^ Bolded items indicate variables that occur at least 2 times across the 3 models: CBC, urinalysis, flu test, IV fluids, pregnancy test, age, CT scan, any imaging, urine culture

# **Appendix Figure 1: Calibration plots, primary analysis**

**Age and sex covariates; Logistic regression**


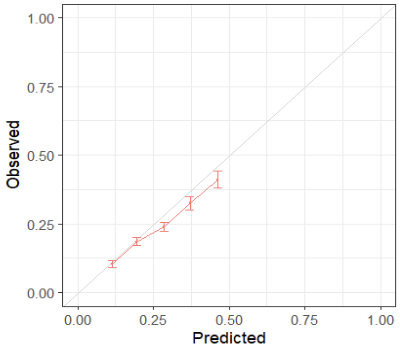


**Age and sex covariates; Random Forest**

**
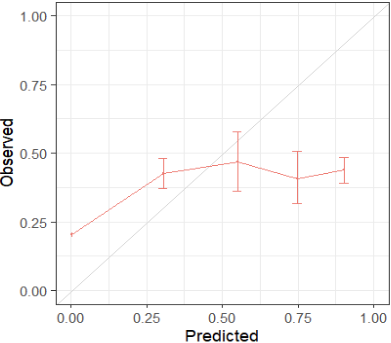
**

**Age and sex covariates; XGBoost**

**
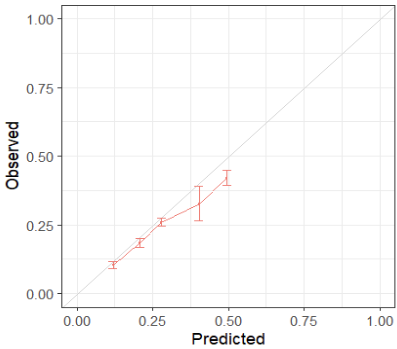
**

**ICD-10 covariates; Logistic regression**


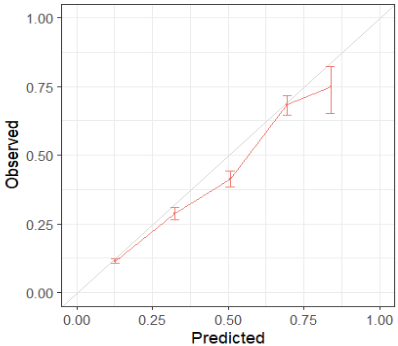


**ICD-10 covariates; Random Forest**

**
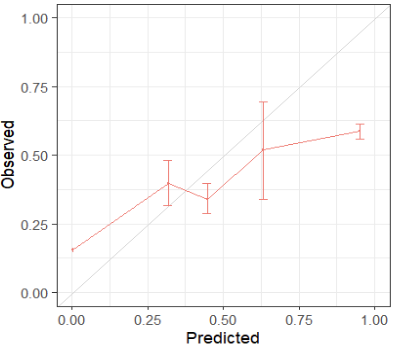
**

**ICD-10 covariates; XGBoost**

**
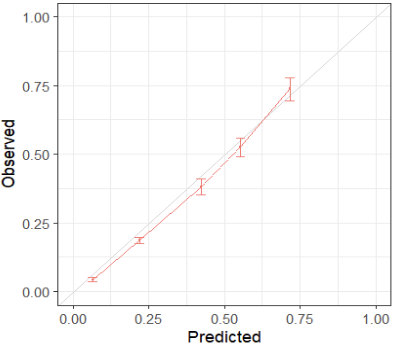
**

**Appendix Figure 1: Calibration plots, primary analysis, cont.**

**ICD-10, age, and sex covariates; Logistic regression**


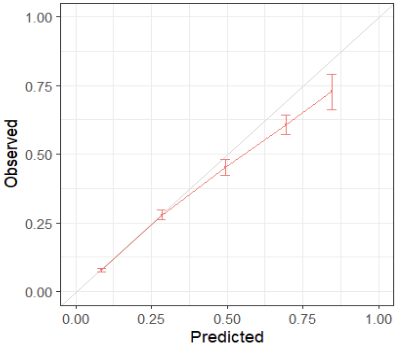


**ICD-10, age, and sex covariates; Random Forest**

**
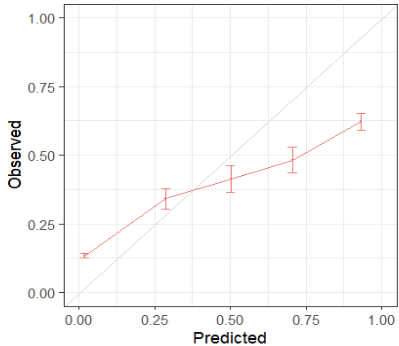
**

**ICD-10, age, and sex covariates; XGBoost**

**
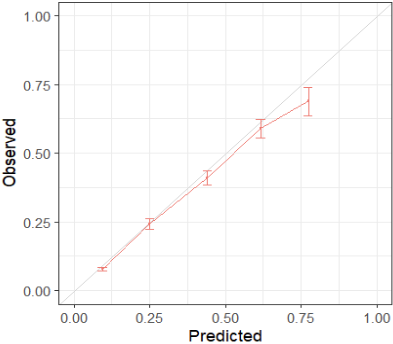
**

**All covariates; Logistic regression**


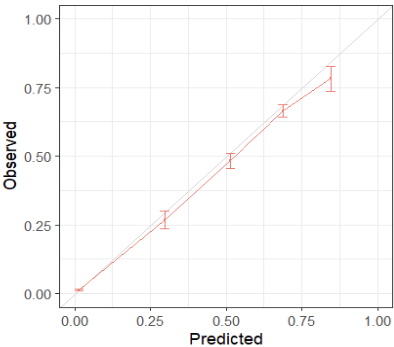


**All covariates; Random Forest**

**
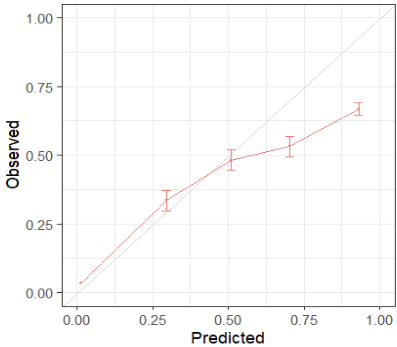
**

**All covariates; XGBoost**

**
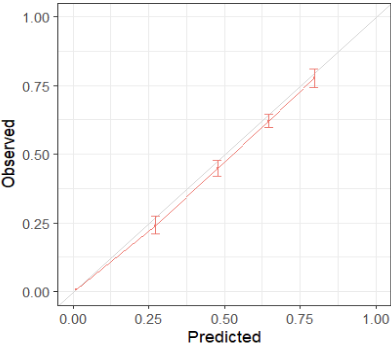
**

**Appendix Figure 1: Calibration plots, primary analysis, cont.**

**Influential subset, Logistic regression**


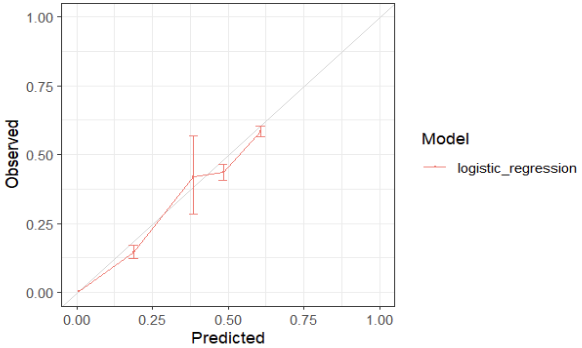


**Influential subset, XGBoost**

**
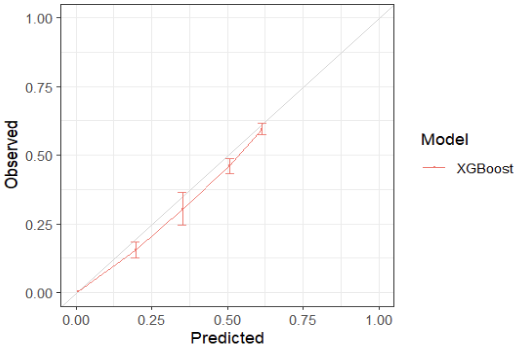
**

**Influential subset, Random Forest**

**
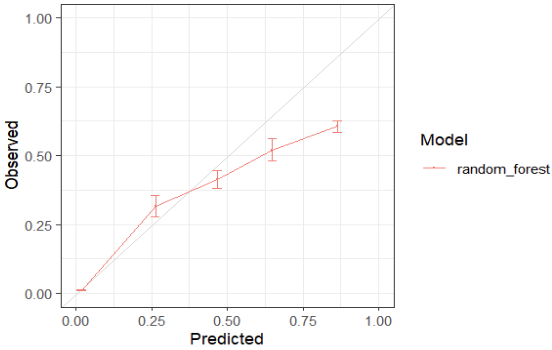
**

# **Appendix Table 5. Model Performance with varying classification thresholds**

| **Model** | **Classification threshold** | **PPV, 95% CI** | **Sensitivity, 95% CI** |
| --- | --- | --- | --- |
| LAVA-L | 0.8 | 0.78 [0.74, 0.83] | 0.12 [0.11, 0.14] |
| LAVA-L | 0.5^a^ | 0.64 [0.62, 0.66] | 0.79 [0.77, 0.81] |
| LAVA-L | 0.3 | 0.58 [0.56, 0.59] | 0.93 [0.92, 0.94] |
| LAVA-X | 0.8 | 0.82 [0.77, 0.87] | 0.09 [0.08, 0.10] |
| LAVA-X | 0.5 | 0.64 [0.62, 0.66] | 0.78 [0.76, 0.80] |
| LAVA-X | 0.3 | 0.57 [0.55, 0.59] | 0.94 [0.93, 0.95] |
| LAVA-L-sub | 0.6^b^ | 0.60 [0.57, 0.62] | 0.42 [0.40, 0.44] |
| LAVA-L-sub | 0.5 | 0.57 [0.55, 0.58] | 0.81 [0.79, 0.82] |
| LAVA-L-sub | 0.3 | 0.54 [0.52, 0.56] | 0.94 [0.93, 0.95] |
| LAVA-X-sub | 0.6 | 0.60 [0.58, 0.63] | 0.48 [0.46, 0.50] |
| LAVA-X-sub | 0.5 | 0.56 [0.54, 0.58] | 0.85 [0.83, 0.86] |
| LAVA-X-sub | 0.3 | 0.54 [0.52, 0.55] | 0.94 [0.93, 0.95] |

Abbreviations:

- LAVA-L, Low Acuity Validation Algorithm – Logistic regression
- LAVA-X, Low Acuity Validation Algorithm – XGBoost
- LAVA-L-sub, Low Acuity Validation Algorithm – Logistic regression trained on the “influential subset” of nine variables (age, any imaging, CBC, CT scan, flu test, IV fluids, pregnancy test, urinalysis, and urine culture)
- LAVA-X-sub, Low Acuity Validation Algorithm – XGBoost trained on the “influential subset” of nine variables (age, any imaging, CBC, CT scan, flu test, IV fluids, pregnancy test, urinalysis, and urine culture)

^a^ Default threshold used in analyses; full results reported in Table 2 of the manuscript

^b^ LAVA-L-sub and LAVA-X-sub had maximum probability predictions for low acuity status of 0.68 and 0.70, respectively. Meaningful interpretation of results is limited at a 0.70 probability threshold or above.

# **Appendix Table 6. Sensitivity analysis, model performance on the validation sample**

| Model | Covariate Set | Sensitivity, 95% CI | Specificity, 95% CI | PPV, 95% CI | NPV, 95% CI |
| --- | --- | --- | --- | --- | --- |
| Logistic regression | Demographics^a^ | 0.03 [0.02, 0.04] | 0.99 [0.98, 0.99] | 0.39 [0.31, 0.47] | 0.78 [0.77, 0.79] |
|  | ICD^b^ (*unchanged)* | 0.37 [0.35, 0.39] | 0.92 [0.92, 0.93] | 0.58 [0.56, 0.61] | 0.83 [0.83, 0.84] |
|  | ICD and demographics | 0.38 [0.36, 0.41] | 0.92 [0.91, 0.93] | 0.58 [0.56, 0.61] | 0.84 [0.83, 0.85] |
|  | Influential subset^c^ | 0.80 [0.79, 0.82] | 0.82 [0.81, 0.83] | 0.56 [0.55, 0.58] | 0.94 [0.93, 0.94] |
|  | All variables | 0.79 [0.77, 0.81] | 0.87 [0.86, 0.88] | 0.64 [0.62, 0.66] | 0.93 [0.93, 0.94] |
| Random Forest | Demographics | 0.10 [0.08, 0.11] | 0.96 [0.96, 0.97] | 0.43 [0.38, 0.47] | 0.79 [0.78, 0.79] |
|  | ICD (*unchanged)* | 0.37 [0.35, 0.39] | 0.92 [0.92, 0.93] | 0.58 [0.56, 0.61] | 0.84 [0.83, 0.84] |
|  | ICD and demographics | 0.43 [0.41, 0.45] | 0.90 [0.89, 0.90] | 0.55 [0.52, 0.57] | 0.85 [0.84, 0.85] |
|  | Influential subset | 0.73 [0.71, 0.74] | 0.85 [0.84, 0.86] | 0.58 [0.56, 0.60] | 0.91 [0.91, 0.92] |
|  | All variables | 0.74 [0.72, 0.76] | 0.86 [0.85, 0.87] | 0.61 [0.59, 0.63] | 0.92 [0.91, 0.93] |
| XGBoost | Demographics | 0.11 [0.10, 0.12] | 0.96 [0.95, 0.96] | 0.42 [0.37, 0.46] | 0.79 [0.78, 0.80] |
|  | ICD (*unchanged)* | 0.34 [0.32, 0.36] | 0.93 [0.93, 0.94] | 0.60 [0.57, 0.62] | 0.83 [0.82, 0.84] |
|  | ICD and demographics | 0.38 [0.36, 0.40] | 0.92 [0.92, 0.93] | 0.58 [0.56, 0.61] | 0.84 [0.83, 0.84] |
|  | Influential subset | 0.83 [0.81, 0.84] | 0.81 [0.80, 0.82] | 0.56 [0.54, 0.58] | 0.94 [0.94, 0.95] |
|  | All variables | 0.78 [0.76, 0.80] | 0.87 [0.87, 0.88] | 0.64 [0.62, 0.66] | 0.93 [0.93, 0.94] |

Abbreviations: PPV, positive predictive value; NPV, negative predictive value; ICD, International Classification of Disease, XGBoost, extreme gradient boosting.

^a^ Age, sex, and race/ethnicity.

^b^ The set of ICD-10 codes included in any of the original seven algorithms; 306 codes total.9

^c^ Includes age, any imaging, CBC, CT scan, flu test, IV fluids, pregnancy test, and urinalysis. See Methods section for details on variable selection.

# **Appendix Table 7. Sensitivity analysis, ten most influential features affecting model predictions^a^**

| Logistic regression | Random Forest | XGBoost |
| --- | --- | --- |
| 1. **CBC^b^** | 1. **CBC** | 1. **CBC** |
| 1. **Urinalysis** | 1. **Age** | 1. **IV fluids** |
| 1. **Flu test** | 1. **Urinalysis** | 1. **Urinalysis** |
| 1. **IV fluids** | 1. **IV fluids** | 1. **Any imaging** |
| 1. **Pregnancy test** | 1. Sex | 1. **CT scan** |
| 1. Ultrasound | 1. **CT scan** | 1. Urine culture |
| 1. ICD M25 (Other joint disorders) | 1. **Any imaging** | 1. **Pregnancy test** |
| 1. ICD S93 (Dislocation and sprain of joints and ligaments at ankle, foot and toe level) | 1. Race/ethnicity: White | 1. **Age** |
| 1. **Age** | 1. **Flu test** | 1. Toxicology screen |
| 1. X-ray | 1. Race/ethnicity: Black | 1. **Flu test** |

^a^ How variable importance is determined:

- Logistic regression: the absolute value of the t-statistic for each model parameter
- Random forest: removal results in greatest decreases in model accuracy
- XG Boost: greatest relative contribution of each variable

^b^ Bolded items indicate variables that occur at least 2 times across the 3 models: CBC, urinalysis, flu test, IV fluids, pregnancy test, age, CT scan, any imaging

# **Appendix Figure 2. Comparison of primary and sensitivity analysis model performance, PPV**

**
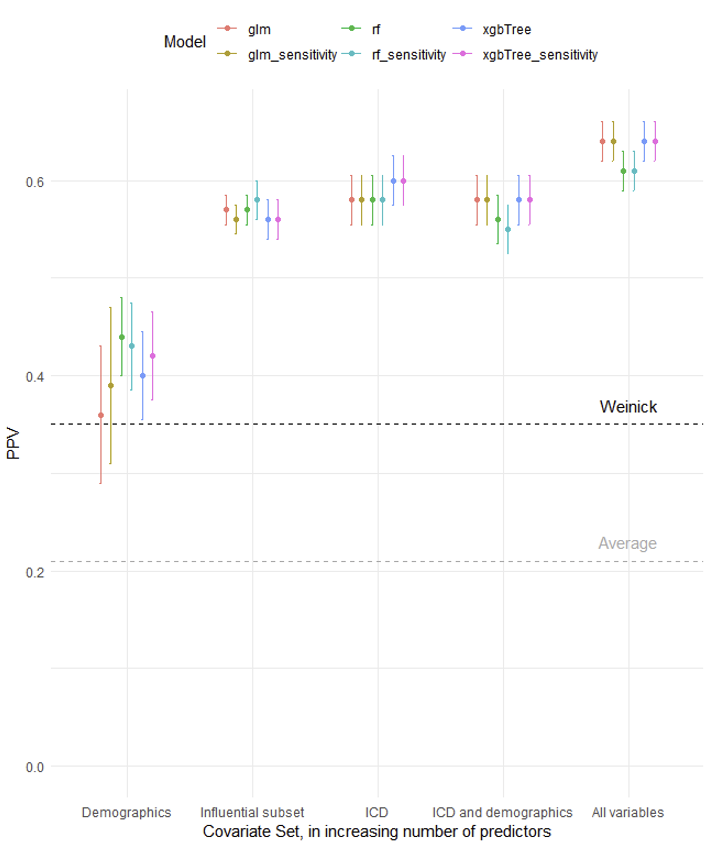
**

# **Appendix Figure 3. Sensitivity analysis, subgroup performance of model trained on all variables (including race/ethnicity)**

**
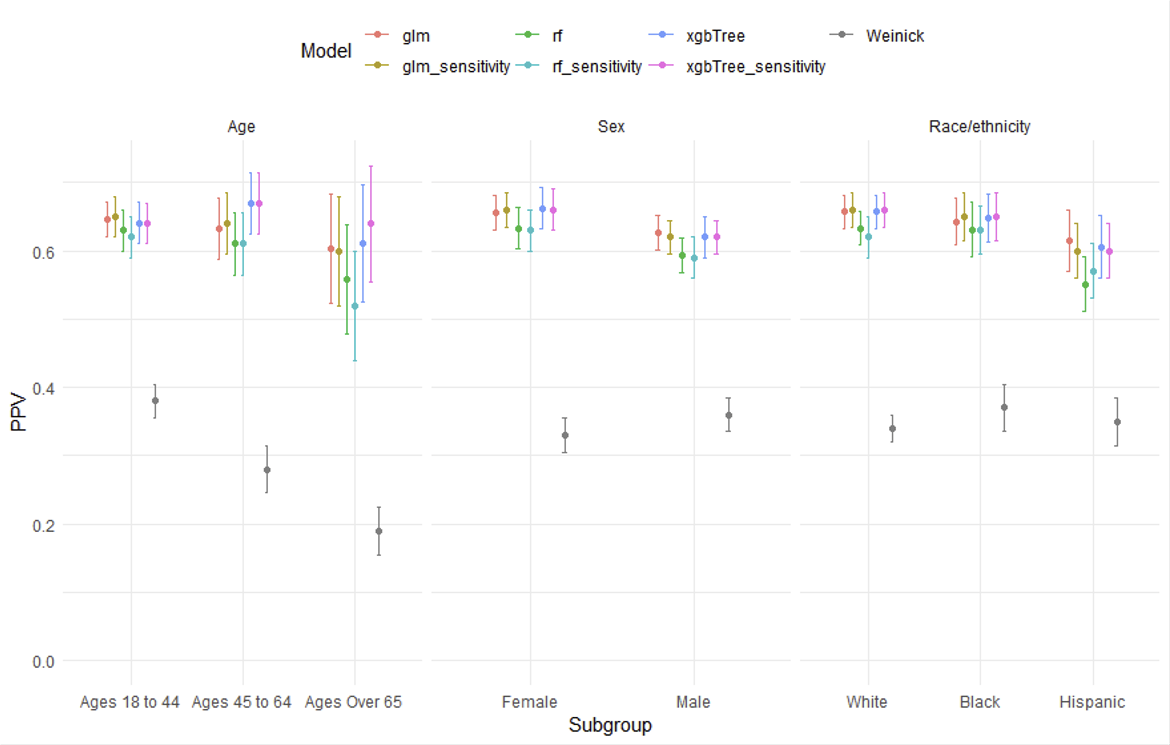
**

# **Appendix Table 8. Model performance on training sample, by model and variable set**

| Model | Covariates | Sensitivity, 95% CI | Specificity, 95% CI | PPV, 95% CI | NPV, 95% CI |
| --- | --- | --- | --- | --- | --- |
| Weinick^a^ | ICD | 0.62 [0.61, 0.63] | 0.67 [0.66, 0.67] | 0.41 [0.40, 0.41] | 0.83 [0.82, 0.83] |
| Average^b^ | ICD | 0.27 [0.12, 0.42] | 0.77 [0.69, 0.84] | 0.26 [0.18, 0.34] | 0.74 [0.72, 0.78] |
| Logistic regression | Age and sex | 0.04 [0.04, 0.04] | 0.98 [0.98, 0.98] | 0.46 [0.45, 0.48] | 0.73 [0.73, 0.74] |
|  | ICD^c^ | 0.40 [0.40, 0.41] | 0.91 [0.90, 0.91] | 0.61 [0.61, 0.62] | 0.80 [0.80, 0.81] |
|  | ICD, age, and sex | 0.43 [0.43, 0.44] | 0.91 [0.90, 0.91] | 0.63 [0.63, 0.64] | 0.81 [0.81, 0.81] |
|  | Influential subset^d^ | 0.80 [0.80, 0.80] | 0.80 [0.80, 0.80] | 0.60 [0.59, 0.60] | 0.92 [0.91, 0.92] |
|  | All variables | 0.78 [0.78, 0.79] | 0.85 [0.85, 0.85] | 0.66 [0.66, 0.67] | 0.91 [0.91, 0.92] |
| Random Forest | Age and sex | 0.16 [0.15, 0.16] | 0.94 [0.94, 0.95] | 0.51 [0.50, 0.52] | 0.75 [0.75, 0.75] |
|  | ICD^c^ | 0.41 [0.40, 0.41] | 0.91 [0.90, 0.91] | 0.61 [0.61, 0.62] | 0.80 [0.80, 0.81] |
|  | ICD, age, and sex | 0.46 [0.45, 0.46] | 0.89 [0.89, 0.89] | 0.61 [0.61, 0.62] | 0.82 [0.81, 0.82] |
|  | Influential subset^d^ | 0.73 [0.73, 0.74] | 0.82 [0.82, 0.82] | 0.60 [0.59, 0.60] | 0.89 [0.89, 0.89] |
|  | All variables | 0.73 [0.73, 0.73] | 0.85 [0.85, 0.85] | 0.64 [0.64, 0.65] | 0.89 [0.89, 0.90] |
| XGBoost | Age and sex | 0.14 [0.13, 0.14] | 0.95 [0.95, 0.95] | 0.52 [0.51, 0.52] | 0.75 [0.75, 0.75] |
|  | ICD^c^ | 0.33 [0.32, 0.33] | 0.93 [0.93, 0.93] | 0.64 [0.63, 0.65] | 0.79 [0.79, 0.79] |
|  | ICD, age, and sex | 0.42 [0.42, 0.43] | 0.91 [0.91, 0.91] | 0.64 [0.63, 0.64] | 0.81 [0.81, 0.81] |
|  | Influential subset^d^ | 0.83 [0.83, 0.83] | 0.79 [0.79, 0.79] | 0.59 [0.59, 0.60] | 0.93 [0.92, 0.93] |
|  | All variables | 0.79 [0.78, 0.79] | 0.85 [0.85, 0.85] | 0.66 [0.66, 0.66] | 0.92 [0.91, 0.92] |

Abbreviations: PPV, positive predictive value; NPV, negative predictive value; ICD, International Classification of Disease, XGBoost, extreme gradient boosting.

^a^ The top-performing algorithm among seven published rule-based algorithms based on ICD-10 codes.

^b^ The average across seven published rule-based algorithms based on ICD-10 codes.

^c^ The set of ICD-10 codes included in any of the original seven algorithms; 306 codes total.

^d^ Includes age, any imaging, CBC, CT scan, flu test, IV fluids, pregnancy test, urinalysis, and urine culture. See Methods section and Appendix Table 4 for details on variable selection.

# **Appendix Table 9. Logistic regression model coefficients**

| Variable | Influential subset | ICD codes | | All variables |
| --- | --- | --- | --- | --- |
| (Intercept) | 0.694 | | -1.471 | 0.519 |
| age | -0.009 | | NA | -0.009 |
| anyimage | -0.431 | | NA | 2.613 |
| bac | NA | | NA | -1.943 |
| bladcath | NA | | NA | 0.015 |
| bloodcx | NA | | NA | -2.583 |
| bnp | NA | | NA | -1.806 |
| bpap | NA | | NA | -0.066 |
| buncreat | NA | | NA | -1.608 |
| cardenz | NA | | NA | -13.863 |
| catscan | -1.339 | | NA | -3.617 |
| cbc | -5.179 | | NA | -4.261 |
| centline | NA | | NA | -0.113 |
| cpr | NA | | NA | -17.491 |
| ctab | NA | | NA | -0.585 |
| ctchest | NA | | NA | -0.564 |
| cthead | NA | | NA | -0.363 |
| ctother | NA | | NA | 0.213 |
| ddimer | NA | | NA | -2.462 |
| edhiv | NA | | NA | -0.399 |
| electrol | NA | | NA | -14.857 |
| endoint | NA | | NA | -15.189 |
| flutest | -1.738 | | NA | -1.720 |
| glucose | NA | | NA | -1.705 |
| hivtest | NA | | NA | -1.888 |
| incdrain | NA | | NA | -0.374 |
| ivfluids | -1.804 | | NA | -1.373 |
| lactate | NA | | NA | -14.204 |
| lft | NA | | NA | -2.770 |
| lumbar | NA | | NA | -1.755 |
| mri | NA | | NA | -3.603 |
| nebuther | NA | | NA | -0.589 |
| othimage | NA | | NA | -2.334 |
| pregtest | -2.090 | | NA | -2.047 |
| pttinr | NA | | NA | -2.591 |
| sex Male | NA | | NA | -0.020 |
| skinadh | NA | | NA | 0.074 |
| suture | NA | | NA | -0.072 |
| toxscren | NA | | NA | -2.467 |
| ultrasnd | NA | | NA | -4.392 |
| urine | -2.118 | | NA | -1.901 |
| urinecx | -2.959 | | NA | -2.808 |
| woundcx | NA | | NA | -0.456 |
| xray | NA | | NA | -3.278 |
| ICD-10 A02 | NA | | -15.095 | -11.262 |
| ICD-10 A04 | NA | | -1.301 | 4.344 |
| ICD-10 A05 | NA | | -15.095 | -18.716 |
| ICD-10 A07 | NA | | -15.095 | -18.108 |
| ICD-10 A08 | NA | | 0.395 | -0.148 |
| ICD-10 A09 | NA | | -0.475 | -0.684 |
| ICD-10 A37 | NA | | 18.037 | 19.067 |
| ICD-10 A38 | NA | | 1.471 | 0.108 |
| ICD-10 A54 | NA | | 0.373 | 0.302 |
| ICD-10 A56 | NA | | 18.037 | 21.764 |
| ICD-10 A63 | NA | | 0.778 | 1.496 |
| ICD-10 A64 | NA | | -0.138 | -0.079 |
| ICD-10 A69 | NA | | -15.095 | -18.263 |
| ICD-10 A74 | NA | | 0.778 | 1.122 |
| ICD-10 B07 | NA | | 2.347 | 1.306 |
| ICD-10 B15 | NA | | -15.095 | -8.817 |
| ICD-10 B16 | NA | | -15.095 | 6.345 |
| ICD-10 B17 | NA | | -15.095 | -11.766 |
| ICD-10 B20 | NA | | -0.831 | -0.855 |
| ICD-10 B30 | NA | | 3.774 | 2.015 |
| ICD-10 B34 | NA | | 0.790 | 0.050 |
| ICD-10 B85 | NA | | 3.263 | 1.869 |
| ICD-10 B86 | NA | | 2.724 | 1.251 |
| ICD-10 B97 | NA | | 0.778 | -0.219 |
| ICD-10 B99 | NA | | 1.877 | 0.433 |
| ICD-10 C44 | NA | | 1.471 | 11.423 |
| ICD-10 D48 | NA | | -15.095 | -19.037 |
| ICD-10 D50 | NA | | -15.095 | -14.472 |
| ICD-10 D53 | NA | | -15.095 | -13.238 |
| ICD-10 D57 | NA | | -3.134 | -2.335 |
| ICD-10 E03 | NA | | -15.095 | -11.400 |
| ICD-10 E04 | NA | | -15.095 | -14.158 |
| ICD-10 E05 | NA | | -15.095 | -12.198 |
| ICD-10 E10 | NA | | -2.499 | -1.615 |
| ICD-10 E11 | NA | | -1.640 | -0.317 |
| ICD-10 E13 | NA | | -15.095 | -16.989 |
| ICD-10 E16 | NA | | -1.541 | 0.017 |
| ICD-10 E51 | NA | | 18.037 | 20.880 |
| ICD-10 E55 | NA | | -15.095 | -13.894 |
| ICD-10 E61 | NA | | -15.095 | -19.855 |
| ICD-10 E66 | NA | | -15.095 | -17.866 |
| ICD-10 E83 | NA | | -15.095 | -15.633 |
| ICD-10 E86 | NA | | -2.768 | -0.895 |
| ICD-10 E87 | NA | | -3.012 | -1.201 |
| ICD-10 E89 | NA | | -15.095 | -11.301 |
| ICD-10 F01 | NA | | -15.095 | -1.473 |
| ICD-10 F03 | NA | | -1.419 | -0.937 |
| ICD-10 F10 | NA | | -1.101 | -0.833 |
| ICD-10 F11 | NA | | -0.344 | -0.997 |
| ICD-10 F32 | NA | | -1.937 | -1.957 |
| ICD-10 F33 | NA | | -15.095 | -17.222 |
| ICD-10 F34 | NA | | -15.095 | -17.658 |
| ICD-10 F39 | NA | | -0.927 | -1.570 |
| ICD-10 F40 | NA | | -15.095 | -13.621 |
| ICD-10 F41 | NA | | -0.162 | -0.640 |
| ICD-10 F43 | NA | | -0.900 | -1.754 |
| ICD-10 F45 | NA | | -0.138 | 2.126 |
| ICD-10 F50 | NA | | 0.778 | 0.213 |
| ICD-10 F60 | NA | | -15.095 | -16.493 |
| ICD-10 G35 | NA | | -0.475 | 1.291 |
| ICD-10 G40 | NA | | -2.040 | -1.875 |
| ICD-10 G43 | NA | | 0.066 | -0.208 |
| ICD-10 G44 | NA | | 0.072 | -0.114 |
| ICD-10 G47 | NA | | 0.717 | 0.116 |
| ICD-10 G56 | NA | | 2.164 | 1.281 |
| ICD-10 G62 | NA | | 0.778 | 0.558 |
| ICD-10 H00 | NA | | 2.570 | 0.988 |
| ICD-10 H01 | NA | | 2.771 | 1.543 |
| ICD-10 H02 | NA | | 1.471 | 0.154 |
| ICD-10 H10 | NA | | 2.721 | 1.423 |
| ICD-10 H11 | NA | | 1.716 | 0.624 |
| ICD-10 H16 | NA | | 2.059 | 0.646 |
| ICD-10 H40 | NA | | 2.164 | 1.611 |
| ICD-10 H60 | NA | | 2.393 | 1.336 |
| ICD-10 H61 | NA | | 2.624 | 1.273 |
| ICD-10 H65 | NA | | 2.439 | 0.869 |
| ICD-10 H66 | NA | | 2.291 | 0.910 |
| ICD-10 H67 | NA | | 1.471 | 14.392 |
| ICD-10 H69 | NA | | 18.037 | 19.431 |
| ICD-10 H91 | NA | | 2.164 | 0.610 |
| ICD-10 H93 | NA | | 2.164 | 1.220 |
| ICD-10 I09 | NA | | -15.095 | 13.461 |
| ICD-10 I10 | NA | | -0.599 | -0.622 |
| ICD-10 I11 | NA | | -1.473 | 0.263 |
| ICD-10 I12 | NA | | -15.095 | -16.567 |
| ICD-10 I13 | NA | | -15.095 | -16.053 |
| ICD-10 I15 | NA | | -15.095 | -17.964 |
| ICD-10 I16 | NA | | -1.473 | 0.532 |
| ICD-10 I20 | NA | | -15.095 | -15.984 |
| ICD-10 I24 | NA | | -15.095 | -15.964 |
| ICD-10 I25 | NA | | -1.787 | -0.297 |
| ICD-10 I49 | NA | | -15.095 | -16.872 |
| ICD-10 I50 | NA | | -3.061 | -0.845 |
| ICD-10 I67 | NA | | -15.095 | -7.023 |
| ICD-10 I70 | NA | | -15.095 | -17.293 |
| ICD-10 I73 | NA | | -1.094 | -0.605 |
| ICD-10 I80 | NA | | 0.698 | 0.851 |
| ICD-10 I82 | NA | | -1.326 | -0.929 |
| ICD-10 I83 | NA | | 0.960 | 1.123 |
| ICD-10 I86 | NA | | -0.608 | -0.503 |
| ICD-10 I87 | NA | | -0.203 | -0.756 |
| ICD-10 I95 | NA | | -2.733 | 0.043 |
| ICD-10 J00 | NA | | 1.694 | 0.598 |
| ICD-10 J01 | NA | | 1.392 | 0.935 |
| ICD-10 J02 | NA | | 1.471 | 0.366 |
| ICD-10 J03 | NA | | 1.299 | 0.300 |
| ICD-10 J04 | NA | | 0.150 | -0.661 |
| ICD-10 J05 | NA | | 1.358 | -0.197 |
| ICD-10 J06 | NA | | 1.456 | 0.432 |
| ICD-10 J09 | NA | | -0.065 | -0.157 |
| ICD-10 J10 | NA | | 0.285 | 0.641 |
| ICD-10 J11 | NA | | 0.672 | 0.516 |
| ICD-10 J12 | NA | | -15.095 | -17.098 |
| ICD-10 J15 | NA | | -0.669 | 0.135 |
| ICD-10 J16 | NA | | -15.095 | -16.936 |
| ICD-10 J18 | NA | | -1.037 | -0.054 |
| ICD-10 J20 | NA | | 0.469 | 0.311 |
| ICD-10 J21 | NA | | 0.397 | -0.537 |
| ICD-10 J30 | NA | | 2.107 | 0.817 |
| ICD-10 J31 | NA | | 1.471 | 0.434 |
| ICD-10 J32 | NA | | 1.242 | 0.911 |
| ICD-10 J34 | NA | | 1.184 | 0.369 |
| ICD-10 J35 | NA | | 2.570 | 0.730 |
| ICD-10 J40 | NA | | 0.036 | -0.156 |
| ICD-10 J41 | NA | | -0.320 | -0.363 |
| ICD-10 J42 | NA | | 0.373 | 0.578 |
| ICD-10 J43 | NA | | -0.475 | 0.250 |
| ICD-10 J44 | NA | | -1.855 | -0.540 |
| ICD-10 J45 | NA | | 0.107 | -0.654 |
| ICD-10 J47 | NA | | -15.095 | 3.556 |
| ICD-10 J81 | NA | | -15.095 | -11.622 |
| ICD-10 J84 | NA | | 0.373 | 1.076 |
| ICD-10 K00 | NA | | 3.417 | 1.475 |
| ICD-10 K01 | NA | | 18.037 | 19.260 |
| ICD-10 K02 | NA | | 2.774 | 1.463 |
| ICD-10 K03 | NA | | 3.263 | 1.487 |
| ICD-10 K04 | NA | | 2.146 | 1.096 |
| ICD-10 K05 | NA | | 2.975 | 1.288 |
| ICD-10 K06 | NA | | 1.066 | 0.537 |
| ICD-10 K08 | NA | | 3.068 | 1.786 |
| ICD-10 K09 | NA | | -15.095 | 9.899 |
| ICD-10 K11 | NA | | 0.190 | -0.136 |
| ICD-10 K12 | NA | | 1.589 | 0.441 |
| ICD-10 K13 | NA | | 2.047 | 1.085 |
| ICD-10 K14 | NA | | 1.471 | 0.531 |
| ICD-10 K21 | NA | | -0.494 | -0.762 |
| ICD-10 K25 | NA | | -0.475 | 7.503 |
| ICD-10 K26 | NA | | -15.095 | -0.759 |
| ICD-10 K27 | NA | | -15.095 | -16.490 |
| ICD-10 K29 | NA | | -1.774 | -1.185 |
| ICD-10 K30 | NA | | -15.095 | -18.018 |
| ICD-10 K31 | NA | | -15.095 | -15.658 |
| ICD-10 K35 | NA | | -15.095 | -15.818 |
| ICD-10 K52 | NA | | -0.262 | -0.136 |
| ICD-10 K57 | NA | | -15.095 | -13.567 |
| ICD-10 K58 | NA | | -15.095 | -16.144 |
| ICD-10 K59 | NA | | 0.430 | -0.035 |
| ICD-10 K70 | NA | | -15.095 | -9.590 |
| ICD-10 L01 | NA | | 3.022 | 1.315 |
| ICD-10 L02 | NA | | 1.588 | 0.945 |
| ICD-10 L03 | NA | | 0.732 | 0.544 |
| ICD-10 L04 | NA | | 0.660 | 0.417 |
| ICD-10 L05 | NA | | 1.851 | 1.450 |
| ICD-10 L08 | NA | | 1.282 | 0.851 |
| ICD-10 L20 | NA | | 2.427 | 2.141 |
| ICD-10 L22 | NA | | 3.486 | 1.520 |
| ICD-10 L23 | NA | | 2.372 | 0.877 |
| ICD-10 L60 | NA | | 3.047 | 1.694 |
| ICD-10 L72 | NA | | 1.551 | 0.447 |
| ICD-10 L89 | NA | | -15.095 | -18.349 |
| ICD-10 L97 | NA | | 0.267 | 0.183 |
| ICD-10 L98 | NA | | 1.411 | 0.623 |
| ICD-10 M10 | NA | | 1.139 | 0.816 |
| ICD-10 M16 | NA | | 0.085 | -0.294 |
| ICD-10 M17 | NA | | 1.471 | 1.218 |
| ICD-10 M19 | NA | | 0.856 | 0.553 |
| ICD-10 M20 | NA | | 1.471 | 0.151 |
| ICD-10 M21 | NA | | 1.066 | 0.483 |
| ICD-10 M25 | NA | | 1.546 | 1.244 |
| ICD-10 M27 | NA | | 1.471 | 2.785 |
| ICD-10 M47 | NA | | -15.095 | -14.273 |
| ICD-10 M53 | NA | | 1.066 | 0.242 |
| ICD-10 M54 | NA | | 0.976 | 0.698 |
| ICD-10 M60 | NA | | 1.877 | 14.058 |
| ICD-10 M62 | NA | | 1.012 | 0.873 |
| ICD-10 M65 | NA | | 1.209 | 1.225 |
| ICD-10 M67 | NA | | 0.219 | -0.248 |
| ICD-10 M70 | NA | | 0.896 | 0.405 |
| ICD-10 M71 | NA | | 1.471 | 1.901 |
| ICD-10 M75 | NA | | 2.047 | 1.320 |
| ICD-10 M76 | NA | | 2.427 | 2.045 |
| ICD-10 M77 | NA | | 2.427 | 1.471 |
| ICD-10 M79 | NA | | 1.002 | 0.906 |
| ICD-10 M99 | NA | | 18.037 | 19.795 |
| ICD-10 N10 | NA | | -2.218 | 1.739 |
| ICD-10 N12 | NA | | -15.095 | -14.711 |
| ICD-10 N13 | NA | | -15.095 | -14.986 |
| ICD-10 N15 | NA | | -15.095 | -13.480 |
| ICD-10 N28 | NA | | -15.095 | -17.270 |
| ICD-10 N30 | NA | | -1.792 | 0.346 |
| ICD-10 N34 | NA | | -1.168 | -1.197 |
| ICD-10 N36 | NA | | -15.095 | -14.915 |
| ICD-10 N39 | NA | | -1.586 | 0.676 |
| ICD-10 N41 | NA | | -15.095 | -14.555 |
| ICD-10 N45 | NA | | -1.301 | -0.502 |
| ICD-10 N48 | NA | | 0.255 | -0.576 |
| ICD-10 N70 | NA | | -15.095 | -3.013 |
| ICD-10 N71 | NA | | -15.095 | -17.757 |
| ICD-10 N72 | NA | | -15.095 | -14.913 |
| ICD-10 N73 | NA | | -1.963 | 0.198 |
| ICD-10 N75 | NA | | 1.001 | -0.103 |
| ICD-10 N76 | NA | | -0.701 | -0.177 |
| ICD-10 N84 | NA | | -15.095 | -8.083 |
| ICD-10 N87 | NA | | -15.095 | 23.819 |
| ICD-10 O23 | NA | | -2.357 | -0.918 |
| ICD-10 O24 | NA | | -15.095 | -15.575 |
| ICD-10 Q34 | NA | | 0.373 | -1.118 |
| ICD-10 Q64 | NA | | -15.095 | -10.540 |
| ICD-10 Q89 | NA | | -0.320 | -1.473 |
| ICD-10 R00 | NA | | -2.159 | -1.491 |
| ICD-10 R04 | NA | | 0.762 | 0.081 |
| ICD-10 R05 | NA | | 0.782 | 0.257 |
| ICD-10 R06 | NA | | -1.432 | -1.240 |
| ICD-10 R07 | NA | | -1.855 | -1.042 |
| ICD-10 R10 | NA | | -2.011 | -0.858 |
| ICD-10 R11 | NA | | -0.091 | 0.004 |
| ICD-10 R21 | NA | | 2.320 | 1.308 |
| ICD-10 R30 | NA | | -0.406 | 1.006 |
| ICD-10 R31 | NA | | -1.825 | 0.830 |
| ICD-10 R32 | NA | | -15.095 | -16.914 |
| ICD-10 R47 | NA | | -15.095 | -17.229 |
| ICD-10 R50 | NA | | 0.643 | 0.183 |
| ICD-10 R51 | NA | | 0.050 | 0.116 |
| ICD-10 R52 | NA | | 0.219 | 0.224 |
| ICD-10 R53 | NA | | -3.010 | -1.616 |
| ICD-10 R56 | NA | | -1.589 | -1.918 |
| ICD-10 R62 | NA | | -15.095 | -18.647 |
| ICD-10 R63 | NA | | -0.566 | -1.260 |
| ICD-10 S00 | NA | | 1.288 | 0.782 |
| ICD-10 S01 | NA | | 1.558 | 0.500 |
| ICD-10 S02 | NA | | 0.683 | 0.671 |
| ICD-10 S03 | NA | | 1.184 | 0.077 |
| ICD-10 S05 | NA | | 1.367 | -0.026 |
| ICD-10 S09 | NA | | 0.771 | 0.171 |
| ICD-10 S10 | NA | | 1.376 | 0.805 |
| ICD-10 S13 | NA | | 1.254 | 0.953 |
| ICD-10 S20 | NA | | 0.835 | 0.507 |
| ICD-10 S21 | NA | | 0.219 | 0.086 |
| ICD-10 S22 | NA | | -0.239 | 0.259 |
| ICD-10 S23 | NA | | 0.912 | 0.436 |
| ICD-10 S29 | NA | | 0.778 | 0.243 |
| ICD-10 S30 | NA | | 1.264 | 1.612 |
| ICD-10 S31 | NA | | 0.460 | -0.136 |
| ICD-10 S32 | NA | | -1.014 | 0.649 |
| ICD-10 S33 | NA | | 1.665 | 1.029 |
| ICD-10 S39 | NA | | 1.441 | 1.066 |
| ICD-10 S40 | NA | | 1.670 | 1.052 |
| ICD-10 S41 | NA | | 1.009 | 0.420 |
| ICD-10 S42 | NA | | 0.676 | 0.090 |
| ICD-10 S43 | NA | | 0.620 | 0.078 |
| ICD-10 S46 | NA | | 1.892 | 1.228 |
| ICD-10 S50 | NA | | 1.811 | 1.239 |
| ICD-10 S51 | NA | | 1.519 | 0.686 |
| ICD-10 S52 | NA | | 0.893 | 0.208 |
| ICD-10 S53 | NA | | 1.665 | 0.283 |
| ICD-10 S56 | NA | | 2.164 | 1.067 |
| ICD-10 S60 | NA | | 2.382 | 1.419 |
| ICD-10 S61 | NA | | 2.119 | 0.994 |
| ICD-10 S62 | NA | | 1.791 | 1.127 |
| ICD-10 S63 | NA | | 2.316 | 1.457 |
| ICD-10 S66 | NA | | 2.090 | 1.410 |
| ICD-10 S70 | NA | | 0.813 | 0.615 |
| ICD-10 S71 | NA | | 1.589 | 0.318 |
| ICD-10 S73 | NA | | 0.373 | 0.125 |
| ICD-10 S76 | NA | | 1.066 | 0.819 |
| ICD-10 S80 | NA | | 1.728 | 1.132 |
| ICD-10 S81 | NA | | 1.755 | 0.797 |
| ICD-10 S82 | NA | | 0.845 | 0.613 |
| ICD-10 S83 | NA | | 1.946 | 1.229 |
| ICD-10 S86 | NA | | 1.534 | 0.935 |
| ICD-10 S90 | NA | | 2.602 | 1.680 |
| ICD-10 S91 | NA | | 2.067 | 1.005 |
| ICD-10 S92 | NA | | 1.678 | 1.044 |
| ICD-10 S93 | NA | | 2.349 | 1.517 |
| ICD-10 S96 | NA | | 2.918 | 2.158 |
| ICD-10 S99 | NA | | 1.902 | 1.071 |
| ICD-10 T14 | NA | | 1.129 | 0.631 |
| ICD-10 T67 | NA | | -15.095 | -17.570 |
| ICD-10 T75 | NA | | 0.883 | 0.666 |
| ICD-10 T78 | NA | | 0.543 | -0.645 |
| ICD-10 T83 | NA | | -0.904 | -1.182 |
| ICD-10 Z01 | NA | | 0.624 | 0.400 |
| ICD-10 Z09 | NA | | 1.471 | 1.031 |
| ICD-10 Z23 | NA | | 3.668 | 2.603 |
| ICD-10 Z29 | NA | | 1.982 | 0.890 |
| ICD-10 Z41 | NA | | 18.037 | 19.317 |
| ICD-10 Z43 | NA | | 0.645 | -0.237 |
| ICD-10 Z47 | NA | | 2.164 | 0.756 |
| ICD-10 Z48 | NA | | 2.818 | 1.531 |
| ICD-10 Z59 | NA | | 0.373 | -0.074 |
| ICD-10 Z63 | NA | | 0.373 | -0.016 |
| ICD-10 Z72 | NA | | -15.095 | -18.628 |
| ICD-10 Z73 | NA | | 1.471 | 1.056 |
| ICD-10 Z74 | NA | | -15.095 | -8.300 |
| ICD-10 Z75 | NA | | 18.037 | 19.464 |
| ICD-10 Z76 | NA | | 2.307 | 1.350 |
